# Surrounding Gastric Mucosa Findings Facilitate Diagnosis of Gastric Neoplasm as Gastric Adenoma or Early Gastric Cancer

**DOI:** 10.1155/2016/6527653

**Published:** 2015-12-24

**Authors:** Tadashi Miike, Shojiro Yamamoto, Yoshifumi Miyata, Tomoya Hirata, Yuko Noda, Takaho Noda, Sho Suzuki, Sachiko Takeda, Shuichiro Natsuda, Mai Sakaguchi, Kosuke Maemura, Kanna Hashimoto, Takumi Yamaji, Hiroo Abe, Hisayoshi Iwakiri, Yoshihiro Tahara, Satoru Hasuike, Kenji Nagata, Akira Kitanaka, Kazuya Shimoda

**Affiliations:** Division of Gastroenterology and Hematology, Department of Internal Medicine, Faculty of Medicine, University of Miyazaki, 5200 Kiyotakecho Kihara, Miyazaki, Miyazaki 889-1692, Japan

## Abstract

*Background and Aim*. It is difficult to master the skill of discriminating gastric adenoma from early gastric cancer by conventional endoscopy or magnifying endoscopy combined with narrow-band imaging, because the colors and morphologies of these neoplasms are occasionally similar. We focused on the surrounding gastric mucosa findings in order to determine how to discriminate between early gastric cancer and gastric adenoma by analyzing the characteristics of the gastric background mucosa. *Methods*. We retrospectively examined 146 patients who underwent endoscopic submucosal dissection for gastric neoplasm between October 2009 and January 2015. The boundary of atrophic gastritis was classified endoscopically according to the Kimura-Takemoto classification system. Of 146 lesions, 63 early gastric cancers and 21 gastric adenomas were ultimately evaluated and assessed. *Results*. Almost all gastric adenomas were accompanied by open-type gastritis, whereas 47 and 16 early gastric cancers were accompanied by open-type and closed-type gastritis, respectively (*p* = 0.037). *Conclusions*. The evaluation of the boundary of atrophic gastritis associated with gastric neoplasms appears to be useful for discrimination between early gastric cancer and gastric adenoma. When gastric neoplasm is present in the context of surrounding localized gastric atrophy, gastric cancer is probable but not certain.

## 1. Introduction

The differential diagnosis of gastric neoplasm as gastric adenoma or early gastric cancer is generally made based on the endoscopic findings and pathological findings obtained from biopsy samples. Because the colors and morphologies of each neoplasm are occasionally similar, it is often difficult to discriminate gastric adenoma from early gastric cancer by conventional endoscopy alone. As for the pathological examination, it is possible that biopsy samples can be obtained only from the adenomatous part of carcinoma in adenoma. Magnifying endoscopy combined with narrow-band imaging (NBI) is reported to be useful for the diagnosis of gastrointestinal tumors, especially early gastric cancers [1–4]. However, it is difficult to master the skill of magnifying endoscopy combined with NBI for the differential diagnosis of gastric adenoma and early gastric cancer, and this method is therefore not practical for everyday use.

Chronic atrophic gastritis, intestinal metaplasia, and* Helicobacter pylori* infection are highly associated with gastric neoplasms [5]. Some patients with chronic atrophic gastritis caused by a* Helicobacter pylori* infection might progress to gastric neoplasm, and patients with chronic atrophic gastritis or* Helicobacter pylori* infection are considered to be at high risk of gastric neoplasm [6, 7]. It is difficult to master the skill of discriminating gastric adenoma from early gastric cancer by conventional endoscopy or magnifying endoscopy combined with narrow-band imaging. However, the relationship between gastric adenoma and early gastric cancer has not yet been clarified. In the present study, we focused on the surrounding gastric mucosa findings in order to determine how to discriminate between early gastric cancer and gastric adenoma by analyzing the boundary of the atrophic gastritis.

## 2. Materials and Methods

### 2.1. Patients

This was a single-center study at the University of Miyazaki Hospital in Japan. We retrospectively examined 146 patients who underwent endoscopic submucosal dissection for gastric neoplasm between October 2009 and January 2015. The study was conducted in accordance with the Declaration of Helsinki and was approved by the Institution Review Board (IRB) of the University of Miyazaki under the ethical guidelines of the Declaration of Helsinki.

### 2.2. Endoscopy Examination

All of the endoscopic examinations were performed and assessed by a single experienced endoscopist (T.M.) with special endoscopic qualifications, using a high-resolution magnifying upper gastrointestinal endoscope (GIF-H260Z, Olympus, Tokyo, Japan) and an electronic endoscopy system (Evis Lucera Spectrum, Olympus, Tokyo, Japan).

### 2.3. Pathological Assessment

All specimens were soaked in formalin solution and routinely processed. Histological diagnoses were performed by experienced pathologists in our hospital. Histology was assessed according to the “Japanese Classification of Gastric Carcinoma” criteria [8].

### 2.4. Assessment of Atrophic Gastritis and Intestinal Metaplasia

**Figure 1:**
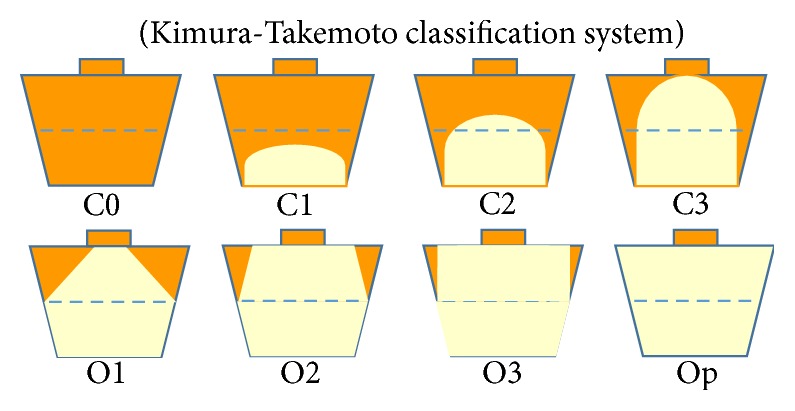
This atrophic border is the boundary between the pyloric and fundic gland regions, which can be recognized endoscopically based on the difference in color and height of the gastric mucosa on either side of the border. There are four types of endoscopic atrophic gastritis: C0-C1, C2-C3, O1-O2, and O3-Op.

**Figure 2:**
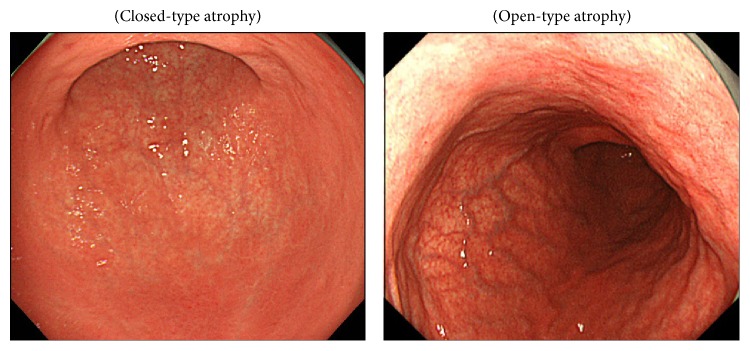


Atrophic gastritis was classified according to the Kimura-Takemoto classification system [9] (Figures 1 and 2), and intestinal metaplasia was also diagnosed endoscopically. If the border of gastric atrophy was on the only lesser curvature of the stomach, it was defined as closed-type (C-type); such cases were subdivided into C0, C1, C2, and C3 patterns as illustrated in Figure 1. If the border was shifted orally and was not limited to the only lesser curvature, it was defined as open-type (O-type); such cases were subdivided into O1, O2, O3, and Op patterns. Therefore, gastric atrophy was typed as C0-l, C2-3, O1-2, or O3-p, based on its boundary.

### 2.5. Diagnosis of* Helicobacter pylori* Infection and Serum Pepsinogen (PG) Levels

Fasting serum samples were taken.* Helicobacter pylori* infection was diagnosed by enzyme immunoassay (EIA) for anti-*H. pylori* immunoglobulin G (IgG). Serum PG levels were determined by chemiluminescent enzyme immunoassay. Patients with serum PG I/II ratio < 3.0 and PG I < 70 ng/mL were defined as having a positive PG test, as proposed by Miki et al. [10–12]. A positive PG test is closely correlated with progression from normal gastric mucosa to extensive atrophic gastritis [13].

### 2.6. Statistical Analysis

Patients were divided into two categories: patients with gastric adenoma and patients with early gastric cancers. The statistical significance of differences in the mean values of patient characteristics was assessed by Student's *t*-test for comparisons between two groups and otherwise by Welch's test. Statistical associations were calculated by univariate analysis comparing the two groups in regard to each item, including the degree of gastric atrophy. Continuous variables were compared by the Mann-Whitney test, and categorical variables were compared by Fisher's exact test. Variables that differed significantly (*p* < 0.05) in the univariate analysis were included in a multivariate analysis based on a logistic regression model to identify which ones were independently related to the result of therapy. Statistical analyses were performed using the statistical software SPSS version 20.0 (SPSS Inc., Chicago, IL, USA), and a *p* value < 0.05 was considered significant.

## 3. Results

### 3.1. Patients and Baseline Characteristics

**Figure 3:**
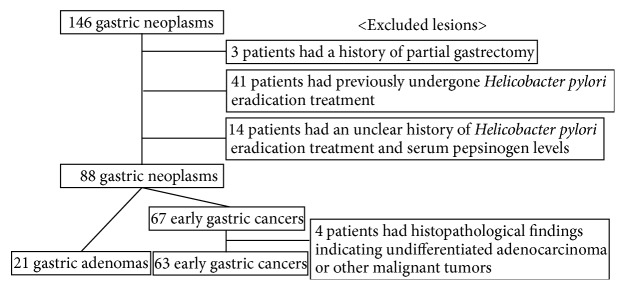


Between October 2009 and January 2015, 146 gastric neoplasms were resected by endoscopic submucosal dissection (ESD). Of the 146 gastric neoplasms enrolled in this study, 3, 41, and 14 were excluded due to the history of partial gastrectomy, previous* Helicobacter pylori* eradication treatment, and unknown history of* Helicobacter pylori* eradication treatment and serum pepsinogen, respectively. A total of 88 gastric neoplasms were examined by histopathologists: 67 were diagnosed as early gastric cancer, and 21 were diagnosed as gastric adenoma. Four gastric neoplasms were excluded based on histopathological findings as undifferentiated adenocarcinoma or other malignant tumors, for example, carcinoma with lymphoid stroma. Ultimately, 63 early gastric cancers and 21 gastric adenomas were evaluated and assessed (Figure 3).

The baseline characteristics of the patients and gastric neoplasms are summarized in Table 1. There was no difference in age or gender between groups. Resection size, resection tumor size, location of tumor, macroscopic types, and presence or absence of ulcer were comparable between the early gastric cancer and gastric adenoma groups.

### 3.2. Endoscopic Gastric Atrophy and Intestinal Metaplasia

Next, we focused on the characteristics of the surrounding gastric mucosa, rather than the gastric neoplasms themselves. All gastric neoplasms were accompanied by endoscopic atrophic gastritis, and its boundary was classified according to the Kimura-Takemoto classification system. Almost all gastric adenomas were accompanied by open-type gastritis, whereas 47 and 16 early gastric cancers were accompanied by open-type and closed-type gastritis, respectively (*p* = 0.037) (Table 2). Intestinal metaplasia was observed in almost all subjects, and there was no difference between groups in the proportion of patients with intestinal metaplasia (*p* = 0.424).

### 3.3. Serum PG Levels and* H. pylori* Antibody

Serum levels of PG I and the ratio of PG I to PG II are associated with the diagnosis of atrophic gastritis, and serum levels of pepsinogen (PGs) are widely used to nonendoscopically screen patients for early gastric cancer [13]. In this study, however, there was no difference in the positivity ratio of serum PG levels between the two groups (*p* = 0.526) (Table 2).* H. pylori* infection is a major cause of chronic gastritis and is reported to be related to early gastric cancer [6, 7]. The positivity rate for* H. pylori* antibodies, however, was not significantly different between the two groups: 76% in the gastric adenoma group and 54% in the early gastric cancer group (*p* = 0.072).

### 3.4. Independent Factors Related to the Probability of Either Gastric Adenoma or Early Gastric Cancer

In the multivariate analysis (Table 2), only the endoscopic gastric atrophy pattern was significantly associated with the diagnosis of either gastric adenoma or early gastric cancer (*p* = 0.006). When the atrophy pattern of the surrounding gastric mucosa was O-type, gastric neoplasms could be either early gastric cancer or gastric adenoma. By contrast, when the atrophy pattern of the surrounding gastric mucosa was C-type, a diagnosis of early gastric cancer was most probable.

## 4. Discussion

To differentiate between gastric adenoma and early gastric cancer using C-WLI (conventional endoscopy with white-light imaging), both the size and color of the gastric neoplasm are useful. Moreover, application of indigo carmine dye also adds some information. The size of the adenoma has some impact on the malignant potential: the malignancy risk of adenomas smaller than 2 cm in diameter ranges from 1% to 5% but increases to more than 50% for larger adenomas [14]. Red coloration is significantly more common in malignant lesion, but its frequency in gastric cancer varied from 60% to 75% [3, 15]. In gastric neoplasms without red coloration, the microvascular and microsurface pattern observed using magnifying endoscopy with narrow-band imaging (M-NBI) is of some utility. In addition to the characteristics of the neoplasm itself, we found that information about the surrounding gastric mucosa serves as a useful reference for differentiating between gastric adenoma and early gastric cancer. All gastric neoplasms are accompanied by gastric atrophy; however, the C-type pattern of endoscopic gastric atrophy was significantly associated with the probability of early gastric cancer.

Correa proposed an ontogeny of gastric cancer throughout the course of the disease, as follows: normal mucosa, superficial gastritis, atrophic gastritis, intestinal metaplasia, dysplasia, and cancer [16]. However, it is accepted that the adenoma–carcinoma sequence is not a major ontogeny of gastric cancer, in contrast to the case of colon cancer, and several reports have shown that intestinal metaplasia does not always reflect precancer status [17, 18]. In our analysis, not only early gastric cancer but also gastric adenoma was highly associated with intestinal metaplasia, and we did not observe a significant difference between gastric adenoma and early gastric cancer in the association with intestinal metaplasia. On the other hand, localized gastric atrophy (C-type) of the surrounding gastric mucosa was highly associated with early gastric cancer, whereas extensive gastric atrophy (O-type) was observed in cases diagnosed with either gastric adenoma or early gastric cancer. In other words, gastric cancer is the probable diagnosis for gastric neoplasm with localized gastric atrophy (C-type) in the surrounding gastric mucosa. Our findings are consistent with a previous report showing that gastric adenoma is observed in patients with extensive gastric atrophy, and there is no sign of progression to gastric cancer in these cases [19].

This study has some limitations. First, it was a retrospective study performed in a single center; therefore, our conclusion should be tested in a well-designed randomized controlled prospective study. Second, the study was performed exclusively in Japanese patients, and the results may not apply fully to the Western population, in which rates of* Helicobacter pylori* infection and atrophic gastritis, as well as levels of gastric acid secretion, may differ.

In conclusion, gastric neoplasms are associated with atrophic gastritis. Based on the current findings, evaluation of the boundary of atrophic gastritis appears to be useful for discriminating between early gastric cancer and gastric adenoma. When gastric neoplasm is present in the context of localized gastric atrophy in the surrounding tissue, gastric cancer is probable but not certain.

## Figures and Tables

**Table 1:** Clinical characteristics of patients.

	Gastric adenoma (*n* = 21)	Early gastric cancer (*n* = 63)	*p* value
Age, years, median (range)	69 (58–88)	72 (50–89)	0.635
Gender (male/female)	13/8	44/19	0.500
Resection size, mm, mean ± SD	37.0 ± 8.6	39.7 ± 12.1	0.261
Resection tumor size, mm, mean ± SD	15.9 ± 1.5	17.7 ± 1.5	0.407
Location of tumor (U/M/L)	6/7/8	13/22/28	0.741
Macroscopic types (I/IIa/IIb/IIc)	0/12/1/8	1/15/1/46	0.079
Ulcer finding (absence/presence)	1/20	6/57	0.494

**Table 2:** Histological outcomes and analysis.

	Gastric adenoma (*n* = 21)	Early gastric cancer (*n* = 63)	Gastric adenoma versus early gastric cancer		
			Univariate analysis	Multivariate analysis	
			*p*	OR (95% CI)	*p*
Intestinal metaplasia (+/−)	19/2	60/3	0.424		0.375

Pepsinogen (+/−)	13/8	34/29	0.526		0.500

*Helicobacter pylori (Hp)* antibody value (+/−)	16/5	34/29	0.072		0.084

Gastric atrophy (C0-C1/C2-C3/O1-O2/O3-Op)	0/1/11/9	0/16/37/10	0.037	3.43 (1.43–8.22)	0.006
